# Integrins in Cancer Drug Resistance: Molecular Mechanisms and Clinical Implications

**DOI:** 10.3390/ijms26073143

**Published:** 2025-03-28

**Authors:** Yoshinobu Kariya, Michiru Nishita

**Affiliations:** Department of Biochemistry, Fukushima Medical University, 1 Hikarigaoka, Fukushima City 960-1295, Fukushima, Japan

**Keywords:** drug resistance, cancer, integrin, drug transporter, extracellular matrix (ECM), epithelial-to-mesenchymal transition (EMT), cancer stem cell (CSC), tyrosine kinase inhibitor (TKI), PD-L1, glycosylation

## Abstract

It is estimated that between 80 and 90% of mortality in cancer patients is directly or indirectly related to drug resistance. Consequently, overcoming drug resistance represents a significant challenge in the treatment of cancer. Integrins are transmembrane adhesion molecules that facilitate the linkage between the extracellular matrix (ECM) and the cytoskeleton, thereby enabling the activation of various cellular signaling pathways. Integrins are highly expressed in various cancers and contribute to cancer progression through invasion and metastasis. In addition, recent studies have revealed that integrins play a pivotal role in the development of drug resistance in cancer. This review will first provide an overview of integrin function and classification. It then discusses recent advances in understanding how integrins contribute to drug resistance in cancer, with a focus on ECM, drug transporters, the epithelial-to-mesenchymal transition (EMT), cancer stemness, PD-L1, and glycosylation. Finally, the potential applications of integrins as targets for therapeutic agents against drug-resistant cancers are also summarized.

## 1. Introduction

In 2022, the Albert Lasker Basic Medical Award was presented to three scientists, Richard Hynes, Erkki Ruoslathi, and Timothy Springer, in recognition of their contributions to the field of integrin research. This was a milestone in the history of integrin research. Today, 40 years have passed since integrins were first discovered [1]. During that time, many researchers have intensively studied the physiological and pathological roles of integrins. It is now well established that integrins play a pivotal role in regulating cellular functions and are also involved in the pathogenesis of various diseases, including cancers, cardiovascular diseases, inflammatory diseases, fibrosis, autoimmune diseases, and Alzheimer’s disease [2]. This has prompted numerous researchers and pharmaceutical companies to develop drugs that target integrins [3]. To date, drugs targeting the Arg-Gly-Asp (RGD)-binding αIIbβ3 integrin have been developed for the treatment of thrombotic cardiovascular events, αLβ2 for dry eye, and α4β7 and α4β1 integrins for ulcerative colitis, Crohn’s disease, and multiple sclerosis [3,4]. On the other hand, despite great efforts, the development of integrin-targeted therapeutics for cancer has not been successful [4,5].

A significant proportion of tumors demonstrate intrinsic or acquired resistance to anticancer drugs during the course of treatment, and approximately 80–90% of mortality in cancer patients is directly or indirectly related to drug resistance [6]. Therefore, drug resistance is a challenge that must be overcome in cancer treatment. In the tumor microenvironment (TME), the cell adhesion of tumor cells to the surrounding extracellular matrix (ECM), including fibronectin, collagen, laminin, and osteopontin, promotes cell survival and proliferation while simultaneously preventing apoptosis [5,7,8,9]. These effects of cell adhesion are now recognized as a major cause of intrinsic and acquired/adaptive therapy resistance, which is referred to as cell adhesion-mediated drug resistance (CAM-DR) [10]. This has already been established in the field of hematological malignancies, where CAM-DR is primarily based on the integrin-mediated adhesion of tumor cells to the ECM [2,11]. For example, the adhesion of myeloma cells to fibronectin through α4β1 and α5β1 integrins induces resistance to doxorubicin by preventing apoptosis [12]. Consequently, integrins are regarded as a promising avenue for developing strategies to overcome chemotherapy resistance in hematological tumors [2]. Recent studies have shown that CAM-DR also occurs in solid tumors [13], and many studies are beginning to report results (Table 1). Therefore, this review aims to advance our understanding of the role of integrins in anticancer drug resistance in solid tumors and presents a comprehensive overview of recent studies conducted in this field.

## 2. Integrin Function and Classification

**Figure 1:**
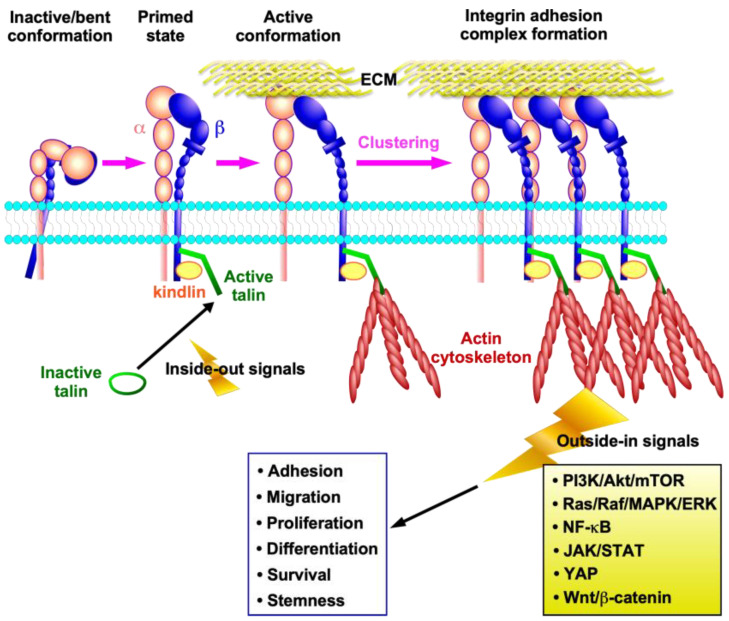
Integrin activation, signaling, and function. Talin activation enables the specific binding of talin to the cytoplasmic tail of the β integrin subunit, which in turn separates the cytoplasmic tails of the αβ subunits, inducing a conformational change in the integrin from a bent conformation to a primed state. Kindlin also activates integrin by strengthening the association between talin and the β subunit tail. Subsequently, the integrin extends its conformation to become activated through binding to the extracellular matrix (ECM) and mechanical forces, thereby promoting integrin clustering and adhesion complex formation. The complex then upregulates various downstream signaling pathways, which ultimately lead to cellular functions.

Integrins are transmembrane adhesion molecules that link the ECM to the intracellular cytoskeleton across the plasma membrane, allowing the transmission of biochemical and mechanical signals between the cell and the extracellular environment (Figure 1). Integrin activation at the plasma membrane is a process that involves a structural conversion [21]. Upon intracellular activation, also known as “inside-out signaling”, talin is recruited to the transmembrane and activated by phosphatidylinositol 4,5-bisphosphate (PIP2). Talin activation enables the specific binding of talin to the β subunit tail, which in turn separates the cytoplasmic tails of the αβ subunits, inducing a conformational change in the integrin from a bent conformation to a primed state (Figure 1). This allows the integrin to become activated and form a connection with the actin cytoskeleton. The binding of kindlin to the β subunit tail facilitates the talin-mediated conformational change in the integrin by inducing a stronger interaction between talin and the β subunit tail, thereby activating it [22]. Subsequently, the integrin assumes an extended conformation through ligand binding and mechanical forces, thereby promoting clustering and integrin adhesion complex formation [2,23] (Figure 1). Integrin engagement with the ECM initiates downstream signaling, which is referred to as “outside-in” signaling. Therefore, integrin functions as bidirectional signal transducers [2,23]. The bidirectional signals regulate a number of processes, including cell adhesion, migration, proliferation, differentiation, survival, stemness, and inflammatory cell activation (Figure 1). These processes are involved in a variety of physiological and pathological processes, including wound healing, inflammation, and cancer.

**Figure 2:**
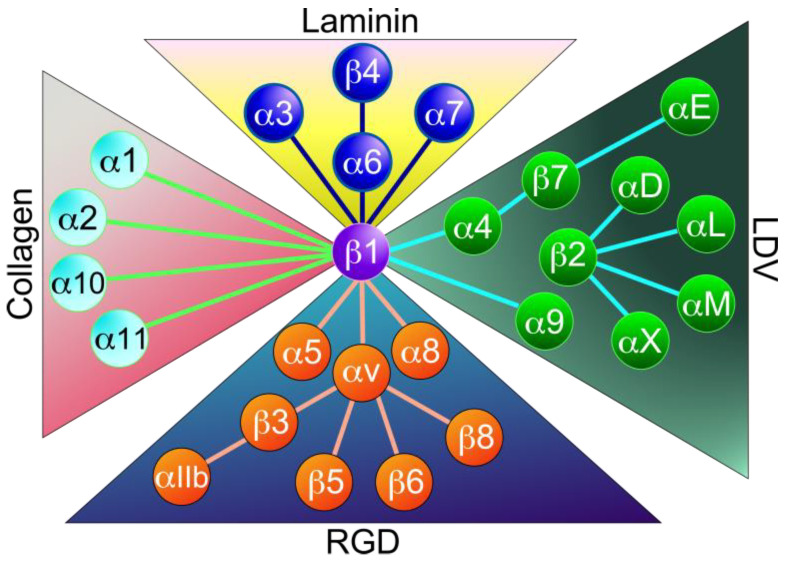
Classification of integrins. Twenty-four integrins are composed of 18α and 8β subunits. They are classified into four major categories: collagen-binding integrins, laminin-binding integrins, LDV-binding integrins, and RGD-binding integrins. This classification is based on ligand binding specificity.

Integrins are heterodimers consisting of a non-covalently bound complex of an α-subunit and a β-subunit (Figure 1). In mammals, a total of 24 different types have been identified, consisting of 18 α and 8 β subunits (Figure 2). Both subunits are classified as type I transmembrane proteins, consisting of a large extracellular domain, a single transmembrane domain, and a short (~30–40 amino acids) intracellular domain (except for β4 integrin, >1000 amino acids). Each integrin has different binding properties to its ligand and is classified into four major types based on these properties: laminin-binding integrins, collagen-binding integrins, RGD-binding integrins, and Leu-Asp-Val (LDV)-binding integrins (Figure 2). α3β1, α6β1, α7β1, and α6β4 integrins are highly specific for laminin receptors. The four collagen-binding integrins (α1β1, α2β1, α10β1, and α11β1) recognize the triple-helical Gly-Phe-Hyp-Gly-Glu-Arg (GFOGER) sequence in the major collagens [2]. Five αv integrins (αvβ1, αvβ3, αvβ5, αvβ6, and αvβ8) and the α5β1, α8β1, and αIIbβ3 integrins recognize the RGD sequence on the proteins such as fibronectin, vitronectin, fibrinogen, osteopontin, and transforming growth factor-β (TGF-β) [2,9]. The LDV-binding integrins bind to an acidic motif related to LDV. This group includes α9β1, αEβ7 integrins, four β2 integrins (αDβ2, αLβ2, αMβ2, and αXβ2), and two α4 integrins (α4β1 and α4β7). The integrins α4β1, α4β7, α9β1, and αEβ7 bind to the LDV sequence, while the β2 integrins recognize a consensus ‘L/I-D/E-V/S/T-P/S’ sequence [24].

## 3. Integrin Expression in Tumors

Integrin expression is regulated by several transcription factors that bind directly to their respective promoter regions. Some integrin subunits in cancer cells are positively regulated by a variety of transcription factors, including MYC (α1, α6, and β4), ZEB1 (α1, α3, and β1), AP-1 (α2, α5, and β6), Ets (α3), Yap1 (α3), PTHrP (α5), ZEB2 (α5), Twist1 (α5), SP1 (α6 and αV), YB-1 (α6), FOXC1 (α7), Ets-1 (β6), FOXO3a (β1), HOXD3 and HOXD5 (β3), E2F (β4), FOSL1 (β4), KLF4 (β4), TAp63 and TAp73 (β4), ZKSCAN3 (β4), Smad3 (β6), and STAT-3 (β6) [25,26,27,28,29,30,31]. In contrast, NFAT1, RREB1, and p53 repress α6, α7, and β4 integrin subunits, respectively. MYC was found to inhibit the transcription of α3, α6, α7, αV, β1, and β3 integrin subunits [25,28].

RNA-seq datasets of integrin genes from 17 human tumor tissues revealed that a variety of integrins are overexpressed or underexpressed in cancer cells [32] (Table 2). α2 (7/17), α3 (7/17), α4 (7/17), α5 (6/17), α6 (9/17), α11 (7/17), αD (6/17), αL (7/17), αM (7/17), αv (7/17), αX (10/17), β1 (6/17), β2 (7/17), β4 (10/17), β5 (8/17), β6 (6/17), β7 (7/17), and β8 (7/17) integrin subunits were significantly upregulated in more than 6 tumor tissues in 17 tumor tissues compared to normal tissues. In contrast, α1 (9/17), α8 (11/17), and α9 (13/17) integrin subunits were more highly expressed in normal tissues than in tumor tissues [32] (Table 2).

Of the 26 integrin subunits, 12 were highly upregulated in breast invasive carcinoma, 21 in cholangiocarcinoma, 13 in glioblastoma, 15 in head and neck squamous cell carcinoma (HNSCC), 15 in kidney renal cell carcinoma, 13 in liver hepatocellular carcinoma (HCC), 9 in lung adenocarcinoma, and 16 in stomach adenocarcinoma compared to normal tissue. In contrast, 11 integrin subunits were downregulated in breast-invasive carcinoma, 15 in kidney chromophobe, 10 in lung adenocarcinoma, 19 in lung SCC, and 16 in prostate adenocarcinoma compared to normal tissue [32].

The following integrins are overexpressed compared to normal tissue: α2β1 integrin in endocervical adenocarcinoma, cholangiocarcinoma, and liver HCC; α3β1 integrin in cholangiocarcinoma, HNSCC, and KIRP; α4β1 integrin in glioblastoma; α5β1 integrin in cholangiocarcinoma, glioblastoma, and HNSCC; α6β1 integrin in cholangiocarcinoma, HNSCC, liver HCC, pancreatic adenocarcinoma (PAAD), and paraganglioma and pheochromocytoma; α11β1 integrin in lung adenocarcinoma and rectum adenocarcinoma; αvβ3 integrin in glioblastoma and lung adenocarcinoma; α6β4 integrin in cholangiocarcinoma, HNSCC, liver HCC, lung adenocarcinoma, lung SCC, PAAD, and stomach adenocarcinoma; αvβ6 integrin in endocervical adenocarcinoma, cholangiocarcinoma, glioblastoma, and HNSCC; and αvβ8 integrin in cholangiocarcinoma, glioblastoma, and lung SCC [32]. In contrast, α8β1, α9β1, and α10β1 integrins were expressed at low or undetectable levels in most cancers. Notably, in both the RNA-seq data and proteomics data, β3 integrin subunit levels were lower or unchanged in most tumor tissues compared to normal tissues. However, it should be noted that integrin expression patterns change at different stages of cancer. For example, the α2β1 integrin increased primary tumor growth and dissemination to bone of the breast cancer cell line MDA-MB231, but α2β1 integrin expression decreased in the bone metastatic clone of MDA-MB231 cells, suggesting that α2β1 integrin expression is increased at the primary tumor growth and dissemination stage but is attenuated at the bone metastatic stage [33]. In addition, integrin expression is altered by treatment stimuli as described in Section 4.2.

## 4. Role of Integrins in Cancer Drug Resistance

Recent studies have shown that integrin plays a pivotal role in the development of drug resistance in solid tumors [34,35]. For example, αvβ3 integrin contributes to cisplatin resistance in osteosarcoma cell line 143B [36] and breast cancer cell lines MDA-MB231 and HCC38 [37]. The linkage of αvβ5 integrin focal adhesions to microtubules by KANK2 confers resistance to microtubule poisons, including paclitaxel and vincristine, in melanoma cell line MDA-MB435S [38]. Moreover, αvβ8 integrin induces resistance to mitomycin C and hydroxycamptothecin in Biu87 and T24 bladder cancer cell lines through Y-box binding protein 1-mediated activation of NF-κB/BCL2 signaling [39]. These observations suggest that various integrins are associated with drug resistance in different types of cancer cells.

**Figure 3:**
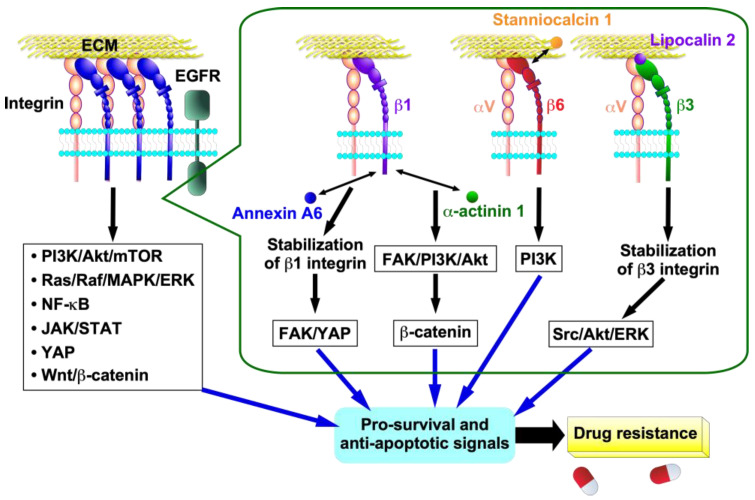
Cellular signalings and mechanisms of integrin-mediated drug resistance in cancer cells. Activated integrins promote multiple intracellular signaling pathways in cancer cells. Interaction of β1 integrin with annexin A6 secreted by cancer-associated fibroblasts (CAFs) stabilizes β1 integrin on the cell surface of cancer cells, which activates the FAK/YAP signaling pathway. Interaction of α-actinin 1 with β1 integrin activates β-catenin signaling through the β1 integrin-mediated FAK/PI3K/AKT pathway. Stanniocalcin 1 binds directly to β6 integrin to activate the αvβ6 integrin-mediated PI3K signaling pathway. The interaction of lipocalin 2 with β3 integrin results in increased αvβ3 integrin stability, which in turn triggers the activation of the Src/Akt/ERK signaling pathway. These signals then induce pro-survival and anti-apoptotic signals, resulting in the acquisition of drug resistance in cancer cells.

**Figure 4:**
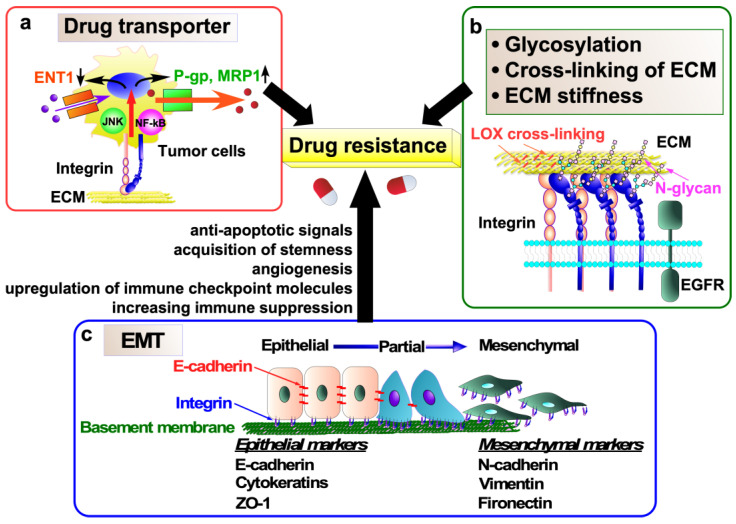
Molecular mechanisms of anticancer drug resistance mediated by integrins. (**a**) Integrins upregulate the expression of drug efflux transporters, including P-gp and MRP1, while simultaneously downregulating the expression of an equilibrative nucleoside transporter, ENT1, in tumor cells. This phenomenon contributes to the development of drug resistance. (**b**) Regulatory mechanisms underlying integrin signaling in tumor cells, which induces drug resistance, encompass integrin *N*-glycosylation, cross-linking of extracellular matrix (ECM) proteins, and ECM stiffness. (**c**) Epithelial-to-mesenchymal transition (EMT) also contributes to cancer drug resistance via induction of anti-apoptotic signaling and angiogenesis, acquisition of stemness, upregulation of immune checkpoint molecules, and increased immunosuppression.

**Figure 5:**
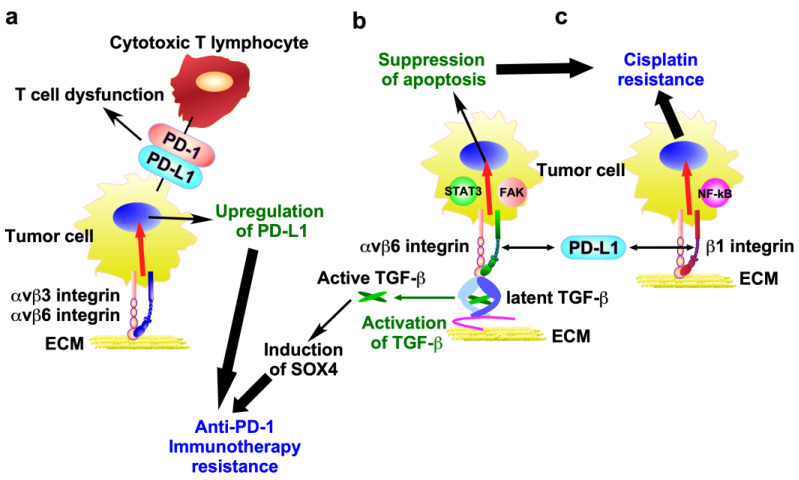
Molecular mechanisms of anti-cancer therapy resistance mediated by integrins and PD-L1. (**a**) αvβ3 and αvβ6 integrins promote PD-L1 expression in tumor cells, thereby facilitating immune surveillance evasion and leading to anti-PD-1 immunotherapy resistance. (**b**) PD-L1 directly interacts with β6 integrin in tumor cells, thereby activating the αvβ6 integrin/FAK/STAT3 signaling pathway, which suppresses cisplatin-induced apoptosis. In addition, αvβ6 integrin activates TGF-β from a latent precursor to induce SOX4 expression, which contributes to anti-PD-1 immunotherapy resistance. (**c**) PD-L1 directly binds to β1 integrin, thereby activating NF-κB signaling and conferring resistance to cisplatin.

This section aims to elucidate the mechanisms through which integrin contributes to drug resistance. To this end, the following topics will be addressed: (1) pro-survival and anti-apoptosis signals, (2) integrin expression induced by drug stimulation, (3) drug transporters, (4) ECM, (5) epithelial-to-mesenchymal transition (EMT), (6) cancer stem cell (CSC) property, (7) tyrosine kinase inhibitors (TKIs), (8) PD-L1, and (9) glycosylation. The relationship between these keywords and integrin-mediated anticancer drug resistance is illustrated in Figure 3, Figure 4 and Figure 5.

### 4.1. Pro-Survival and Anti-Apoptosis Signals

Cancer cells can acquire drug resistance through integrin-mediated pro-survival and anti-apoptosis signals, including PI3K/Akt/mTOR, Ras/Raf/MAPK/ERK, NF-κB, JAK/STAT, YAP, and Wnt/β-catenin [40,41,42] (Figure 3). β1 integrin contributes to cisplatin resistance and tumorigenesis in HNSCC [43]. The overexpression of α-actinin 1 was found to be significantly correlated with a suboptimal response to neoadjuvant chemotherapy and reduced overall survival in patients with HNSCC [43]. The interaction of α-actinin 1 with β1 integrin activates β-catenin signaling through the β1 integrin-mediated FAK/PI3K/AKT pathway, which results in tumorigenesis and cisplatin resistance [43] (Figure 3). Stanniocalcin 1 (STC1) is a secreted glycoprotein hormone that has been detected in various human tissues [44]. Various cancers overexpress STC1, and STC1 promotes chemoresistance to cisplatin by directly binding to αvβ6 integrin to activate the PI3K signaling pathway in ovarian cancer cell lines Skov3-ip1 and Hey [44,45] (Figure 3).

As discussed above, cancer cells express multiple integrins, and some cancer drug resistance is associated with the multiple integrin-mediated pro-survival and anti-apoptosis signals. Dual drug-resistant melanoma cell lines SKMEL28 and WM2664 to MAPK and PI3K/mTOR inhibitors exhibited increased expression of α3β1 and α11β1 integrins [46]. The integrins activate Src-YAP1, thereby conferring dual resistance to MAPK and PI3K/mTOR inhibitors to melanoma cells [46]. These observations suggest that multiple integrins may be involved in regulating resistance to anticancer drugs.

### 4.2. Induction of Integrin Expression by Drug Stimulation

Some integrin expressions are induced by anticancer drug stimulation, resulting in the drug resistance of cancer cells [29,47,48,49,50]. Docetaxel treatment was shown to lead to increased αvβ3 integrin expression in a murine bone metastasis model using the PyMT-BO1 and 4T1 murine breast cancer cell lines [51]. In addition, 97% of triple-negative breast cancer (TNBC) patient samples were β3 positive after chemotherapy. α5β1 integrin expression is significantly upregulated in cisplatin-resistant esophageal SCC cell lines Eca109 and TE-1 and contributes to cisplatin resistance by activating the FAK/PI3K/Akt/BRAD1 signaling pathway [47]. Lenvatinib is a multi-kinase inhibitor approved as a first-line therapy for advanced HCC, but many patients with HCC either do not respond to lenvatinib or later develop resistance [48,49]. Recent reports have shown that αvβ8 integrin expression is elevated in lenvatinib-resistant HCC cell lines Hep3B and Huh7 and the integrin confers lenvatinib resistance in HCC cells via the activation of the HSP90/AKT signaling pathway [48].

Given the prevalence of aberrant expression and the constitutive activation of some transcription factors found in chemoresistant cancers [52], it is conceivable that integrin expression may be regulated at the transcriptional level in chemoresistant cancers [29,30,53]. Indeed, in osteosarcoma cell lines 143B and Saos-2, cisplatin induces the expression of the zinc transporter ZIP10, which increases the expression of α10β1 integrin by activating CREB [53]. The ZIP10-induced α10β1 integrin activates the PI3K/AKT pathway, which confers proliferation and chemoresistance to osteosarcoma [53].

Another regulatory mechanism for increased integrin expression by drug stimulation is increased integrin protein stability. For instance, the interaction of β3 integrin with lipocalin 2, which is overexpressed by 5-FU-treatment, led to increased αvβ3 integrin stability. This, in turn, initiated the activation of the Src/Akt/ERK-mediated anti-apoptotic program, thereby contributing to the development of 5-FU resistance in colorectal cancer HT29 cells [50] (Figure 3). Annexin A6 in extracellular vesicles from cancer-associated fibroblasts (CAFs) is transferred into gastric cancer cells, where it stabilizes β1 integrin at the gastric cancer cell surface [54]. This occurs without any effect on β1 integrin mRNA expression. The stabilized β1 integrin at the cell surface activates the FAK-YAP signaling pathway, which subsequently induces drug resistance (Figure 3). This process can be effectively attenuated by FAK and YAP inhibitors in vitro and in vivo. Circular RNA (circRNA) is a single-stranded, covalently closed, continuous loop structure that lacks free ends and a polyadenylate tail [55]. CircRNAs competitively bind to and sponge miRNAs, thereby stabilizing their target transcripts [55]. CircRNA, which plays an important role in cancer development and progression, also contributes to drug resistance in cancer cells by stabilizing integrin [56,57,58]. For example, circPDSS1 directly interacts with miR-515-5p, which targets ITGA11 and acts as a sponge for miR-515-5p, resulting in the stabilization of α11β1 integrin expression and cisplatin resistance in the gastric cancer cell lines HGC-27 and AGS [57]. In fact, the expression of circPDSS1 and α11β1 integrin was markedly elevated, while that of miR-515-5p was diminished in cisplatin-resistant gastric cancer tissues and cells (HGC-27/DDP and AGS/DDP cells) relative to controls. Intriguingly, circRNF13 indirectly stabilizes β1 integrin mRNA by promoting IGF2BP1-mediated phase separation in the oral cancer cell line CAL-27 [58].

Integrin trafficking is important for the regulation of integrin function, and the Rab and Arf GTPase families are key regulators of integrin trafficking [59]. Rab25 expression is associated with the therapeutic response to epidermal growth factor receptor (EGFR)-TKIs, such as erlotinib, in non-small cell lung cancer (NSCLC) [60]. Rab25 interacts with β1 integrin and facilitates its trafficking into the cytoplasmic membrane, thereby increasing the cell surface expression of β1 integrin and subsequent induction of erlotinib resistance [60]. Taken together, these observations suggest that integrin expression induced by drug stimulation plays a pivotal role in drug resistance in cancer cells.

### 4.3. Drug Transporter

Transporter-associated drug resistance is a major obstacle in cancer therapy [61]. Recent studies have shown that integrin can confer drug resistance to cancer cells by regulating the expression of drug transporters (Figure 4a). α3β1 integrin confers gemcitabine resistance to pancreatic cancer cells by inhibiting the expression of the ENT1 [62]. The nucleotide transporter ENT1 transports gemcitabine, which is widely used as an anticancer chemotherapeutic agent for various solid tumors, into cells [63]. Therefore, high levels of ENT1 predict a good response of pancreatic cancer to gemcitabine treatment, whereas the loss of ENT1 leads to chemical drug resistance [63,64]. α3β1 integrin/JNK signaling inhibits the uptake and accumulation of gemcitabine in pancreatic cancer cells by ultimately inhibiting the expression of the ENT1, although the detailed molecular mechanisms remain unclear [62].

P-glycoprotein (P-gp, also known as MDR1) is encoded by the ATP-binding cassette (ABC) subfamily B member 1 (ABCB1) gene and functions as an energy-dependent efflux pump [61]. It transports a wide range of substrates, including drugs such as colchicine, tacrolimus, and quinidine; chemotherapeutics such as doxorubicin, etoposide, erlotinib, gefitinib, paclitaxel, sorafenib, and vinblastine; and others such as bilirubin, digoxin, dexamethasone, lipids, and steroids. In clinical specimens of various cancers, overexpression of P-gp has been associated with poor response to chemotherapy [61]. P-gp is upregulated by integrins in cancer and excludes anticancer drugs from the cancer cells, leading to drug resistance in cancer cells (Figure 4a). In fact, αvβ6 integrin and β1 integrin upregulate P-gp expression and contribute to doxorubicin and cisplatin resistance in doxorubicin-resistant MCF-7/ADR breast cancer cells and cisplatin-resistant TU686 and TU138 laryngeal SCC cells, respectively [65,66]. Similarly, the αvβ3 integrin/osteopontin interaction increases P-gp expression and develops drug resistance to daunomycin, paclitaxel, doxorubicin, actinomycin-D, and rapamycin in prostate cancer cell line PC-3 [67]. These findings suggest that the discovery of drugs targeting the regulation of drug transporters by integrins may be useful in the treatment of drug-resistant cancers.

### 4.4. Extracellular Matrix (ECM)

The ECM plays a key role in cancer drug resistance by modulating drug delivery and efficacy. For example, the hyaluronan-rich ECM in PDAC not only acts as a physical barrier to drug penetration but also prevents drug perfusion, diffusion, and convection by increasing interstitial fluid pressure and inducing vascular collapse [68].

In recent years, it has become clear that integrins are involved in ECM-mediated cancer drug resistance (Figure 4b). In sorafenib non-responsive patients, elevated α5β1 integrins contribute to sorafenib resistance by promoting the formation of vasculogenic mimicry in HCC [69]. Resistance to sorafenib in HCC is also associated with laminin-332 produced by hepatic stellate cells in the HCC ECM [70]. Sorafenib downregulates total FAK, which then undergoes ubiquitin-mediated proteasomal degradation. The α3β1 integrin/laminin-332 signaling axis promotes the escape of the FAK from ubiquitination, thereby conferring sorafenib resistance. Bone-metastatic castration-resistant prostate cancer (CRPC) is a lethal disease due to the inherent resistance to androgen deprivation therapy, chemotherapy, and targeted therapies [19]. Approximately 70% of CRPC patients exhibit constitutive activation of the PI3K survival pathway, primarily due to PTEN loss. Nevertheless, targeting the PI3K/mTOR pathway has failed to improve overall survival in clinical trials [19]. This is due to the fact that α6β1 integrin-mediated cell adhesion to laminin and hypoxia-induced expression of PIM kinases contribute to resistance to PI3K inhibitors in the PTEN-negative CRPC tumors by reducing oxidative stress and preventing cell death [19].

ECM stiffness in TME contributes to drug resistance by modulating integrin signaling pathways [71,72]. PDAC cell lines, Panc-1, Capan-1, and PDAC-3, growing on stiff substrates (50 kPa) similar to PDAC substrate stiffness, increased α2β1 integrin expression and acquired α2β1 integrin-dependent resistance to gemcitabine [73]. Similarly, increased ECM stiffness (21 kPa) protects ovarian high-grade serous carcinoma cells OVCAR4 and OVCAR8 against cisplatin-induced apoptosis via β1 integrin/FAK/YAP signaling [74]. In chemoresistant TNBC tumors, increased hypoxia/HIF1-α and ECM stiffness downregulate miR-326, which targets the fibronectin receptor, α5 integrin. Consequently, TNBC cells upregulate α5 integrin gene expression, thereby acquiring fibronectin-driven doxorubicin resistance through α5β1 integrin/FAK/Src signaling pathways [75].

The stiffness of ECM is subject to regulation by lysyl oxidase (LOX), a secreted copper-dependent amine oxidase whose primary function is the covalent cross-linking of collagens and/or elastin. Aberrant expression and activity of LOX have been reported in cancer [76]. The increased matrix stiffness induced by LOX has been shown to upregulate α5β1 integrin and fibronectin expression, leading to the activation of FAK/Src signaling, the suppression of apoptosis, and resistance to doxorubicin [77] (Figure 4b).

### 4.5. Epithelial-to-Mesenchymal Transition (EMT)

EMT is a process through which cells transition from a fully epithelial phenotype to various intermediate hybrid states (partial EMT) and, finally, to a fully mesenchymal phenotype (full EMT) [78] (Figure 4c). During the trans-differentiation, cells lose cell polarity and cell–cell adhesion, followed by the acquisition of the motile mesenchymal phenotype via cytoskeletal reorganization [79]. In cancer, EMT, especially partial EMT, contributes to tumorigenesis, invasion, metastasis, stemness, immune evasion, and drug resistance [79]. EMT contributes to therapeutic resistance by reducing the sensitivity to proapoptotic signals, acquiring stemness features, stimulating angiogenesis, upregulating the expression of immune checkpoint molecules, and increasing immune suppression [80]. Recent studies have shown that integrin is involved in the induction of EMT in cancer cells and plays an important role in EMT-mediated chemoresistance [81,82] (Figure 4c).

Blockade of the interaction of αvβ3 integrin with EGF-like repeats and discoidin I-like domains 3 (EDIL3) by a potent inhibitor of αvβ3 integrin, cilengitide, reversed the mesenchymal phenotype and restored the sensitivity to paclitaxel in the paclitaxel-resistant breast cancer cell line MDA-MB231 and prostate cancer cell line PC3. This finding suggests that the interaction of αvβ3 integrin with EDIL3 induces EMT and paclitaxel resistance [83]. Elevated levels of α2β1 integrin have been associated with a poor prognosis in gastric cancer patients who have undergone chemotherapy [84]. In the doxorubicin-resistant gastric cancer cell SGC7901/ADR and the vincristine-resistant gastric cancer cell SGC7901/VCR, miR-135b-5p, a direct upstream regulator of α2β1 integrin, is downregulated, thereby increasing α2β1 integrin expression. The α2β1 integrin induces chemoresistance and antiapoptotic effects by activating MAPK signaling and inducing EMT [84]. Similarly, in gefitinib-resistant EGFR-mutant NSCLC cell lines (HCC827GR and PC9GR), decreased expression of miR-483-3p, which is caused by hypermethylation of its promoter, upregulates αvβ3 integrin expression and the subsequent activation of the FAK/ERK pathway, thereby promoting EMT and subsequent acquired gefitinib resistance [85].

Neutrophil extracellular traps (NETs) are net-like structures composed of DNA-histone complexes and proteins released by activated neutrophils [86]. Chemotherapy induces neutrophil recruitment and the secretion of IL-1β from cancer cells, which in turn triggers NET formation [87]. NETs activate latent TGF-β via αvβ1 integrin and MMP-9, leading to EMT and chemoresistance.

### 4.6. Cancer Cell Stemness

CSCs are the cancer cells that possess the unique properties of self-renewal and multilineage differentiation, which are responsible for tumor initiation and intertumoral heterogeneity [88,89]. CSCs evade the anti-proliferative effects of anticancer drugs by slowing down their growth [88]. In addition, CSCs overexpress ABC drug transporters [41]. Thus, CSCs are thought to contribute to resistance to anticancer therapy.

αvβ3, α6β1, and α6β4 integrins have been shown to function as positive regulators of normal and cancer stemness [34,81,82,90,91]. For example, the binding of αvβ3 integrin to the fibronectin-rich ECM has been shown to confer stemness properties to the pancreatic cancer cell lines 34E, 79E, and Colo-357 [92]. Mesenchymal-like breast cancer stem cells deposit laminin-511 and thereby promote cancer self-renewal and tumor initiation through the interaction of α6Bβ1 integrin with laminin-511 and the subsequent activation of the Hippo transducer TAZ [93]. α6β4 integrin has been shown to increase the number of ITGB4^high^ CSCs in residual tumors and the tumor-initiating capacity of the murine mammary carcinoma cell line 4T1 and the HNSCC cell line SCC7 [91].

Recent studies have demonstrated that other types of integrins can also regulate stemness. For example, α5β1 integrin and αvβ8 integrin have been shown to play a critical role in maintaining CSC properties in CD44^+^ EpCAM^+^ CSCs isolated from gastric cancer cell line AGS [94] and in primary glioblastoma stem cells [95], respectively. In addition, α7β1 integrin regulates the stemness of HCC cell lines SMMC-7221 and Hep G2 and esophageal SCC cell lines KYSE180 and KYSE520 by activating the PTK2-PI3K/Akt and FAK signaling pathways [96,97]. Furthermore, the interaction of α2β1 integrin with collagen has been demonstrated to enhance metastasis and stemness through the activation of the PI3K/Akt/Snail signaling pathway in colorectal cancer cell line HCT-116 [98].

Indeed, integrin-mediated cancer stemness is associated with drug resistance. For instance, the resistance of MDA-MB231 (breast), A549 (lung), and Panc-1 (pancreas) cancer cells to the EGFR inhibitor erlotinib is associated with αvβ3 integrin-mediated cancer stemness [99]. α6β4 integrin epigenetically upregulates the expression of the stemness marker SOX2 through histone acetylation at the Sox2 promoter site, thereby conferring cisplatin resistance in lung cancer cell lines H520 and SBC5 [100]. The α6β4 integrin signaling cascade, which includes FAK, Src, and ERK, as well as stabilization and nuclear translocation of β-catenin, is activated by the interaction of keratin 17 with plectin in oral SCC (OSCC) cell lines C9IV3 and HSC3 [101]. The activation of this signaling cascade is associated with increased cancer stemness and cisplatin resistance in the OSCC cell lines [101].

### 4.7. Tyrosine Kinase Inhibitor (TKI) Resistance

It has been estimated that only approximately 30% of patients can benefit from TKIs. However, this population often develops drug resistance within six months [102]. Sorafenib, a multikinase inhibitor, targets the Raf serine/threonine kinases and receptor tyrosine kinases, including vascular endothelial growth factor receptor (VEGFR)-2, VEGFR-3, platelet-derived growth factor receptor (PDGFR)-β, c-KIT, and Flt-3 [103]. Sorafenib exerts antitumor effects on recurrent tumors in HCC. However, the aberrant expression of αvβ5 integrin in HCC cell lines Huh7 and Hep3B decreases the sensitivity of HCC cells to sorafenib [102]. αvβ5 integrin increases the protein level of casein kinase 1 α1 through the PI3K/AKT pathway, resulting in the upregulation of phosphorylation of αvβ5 integrin, which enhances the interaction between αvβ5 integrin and EGFR pathway substrate 15 (EPS15). This, in turn, activates the EGFR in HCC cells, thereby contributing to the reduced sensitivity of HCC cells to TKIs.

EGFR-TKIs, including gefitinib, erlotinib, afatinib, and dacomitinib, represent the primary therapeutic option for advanced NSCLC cases with *EGFR* mutations [104]. However, the majority of patients with EGFR-mutant NSCLC develop resistance to EGFR-TKIs after several months of the treatment [104]. One of the major causes of this is the upregulation of αvβ3 integrin by osteopontin, which contributes to acquired gefitinib resistance in NSCLC cell lines PC9 through the activation of the FAK/AKT and ERK pathways [40]. Moreover, αvβ3 integrin plays a pivotal role in mediating resistance to HER2 and EGFR-TKI neratinib-induced ferroptosis through the reprogramming of the iron/antioxidant metabolism and sustained activation of Akt signaling in murine HER2-positive brain metastatic breast cancer cell line TBCP-1 [105].

αvβ3 integrin also contributes to TGF-β1-mediated EGFR-TKI resistance in EGFR-mutant lung cancer. Osimertinib, a third-generation of EGFR-TKI, has been shown to significantly and sustainably increase the expression of both TGF-β1 and αvβ3 integrin in in vitro and in vivo models of EGFR-mutant lung cancer (HCC827 NSCLC cells) with acquired resistance to osimertinib [106]. The interplay between TGF-β1 and αvβ3 integrin forms a positive feedback loop, leading to increased expression of both proteins and activated downstream signaling pathways, including αvβ3 integrin/FAK/Src/ERK and TGF-β1/Smad. The combination therapy involving the blockade of TGF-β1 and αvβ3 integrin using specific inhibitors, SB431542 and cyclo (-RGDfK), resulted in a significantly higher rate of apoptosis in osimertinib-resistant HCC827 cells compared to the use of a single agent alone [106]. These results suggest that integrin signaling regulates TKI resistance.

### 4.8. PD-L1

Immune checkpoint molecules are inhibitory receptors expressed on immune cells that suppress immune activation when binding to specific ligands. Tumor cells exploit the immune checkpoint system to establish an immunosuppressive state that facilitates immune surveillance evasion and tumor growth [9]. To illustrate, the interaction of programmed cell death ligand 1 (PD-L1) on tumor cells with programmed cell death protein 1 (PD-1) on cytotoxic T lymphocytes has been demonstrated to induce T cell dysfunction, thereby allowing cancer cells to evade immune surveillance (Figure 5a). Consequently, the inhibition of the binding of PD-L1 to tumor cells with PD-1 can result in the upregulation of lymphocyte cytotoxic activity and the enhancement of the immune response [107]. This strategy has led to the development of immune checkpoint inhibitors, including atezolizumab, pembrolizumab, and nivolumab [107].

αvβ3 and αvβ6 integrins promote PD-L1 expression in ovarian cancer cell line SK-OV3 and colon cancer cell lines HT-29 and WiDr, thereby facilitating immune surveillance evasion [108,109] (Figure 5a). Indeed, this integrin depletion resulted in a reduction in PD-L1 expression in cancer cells, and the combination of β3 integrin depletion and anti-PD-1 led to highly effective immunotherapy. These observations suggest that upregulated integrins lead to a decreased response to anti-PD-1 immunotherapy by promoting PD-L1 expression.

The interaction of integrin with PD-L1 seems to activate integrin signaling related to drug resistance. The direct interaction of PD-L1 with αvβ6 integrin activates the αvβ6 integrin/FAK/STAT3 signaling pathway, which suppresses cisplatin-induced apoptosis in bladder cancer cell lines 5637 and UC3 [110] (Figure 5b). Similarly, PD-L1 directly binds to β1 integrin, thereby activating NF-κB signaling and eliciting a poor response to cisplatin in NSCLC cell lines A549 and PC9 [111] (Figure 5c).

αvβ6 integrin activates TGF-β from a latent precursor to induce SOX4 expression in the human TNBC cell line BT549, which contributes to tumor cell resistance to cytotoxic T cells [112] (Figure 5b). Antibody-mediated inhibition of αvβ6 integrin induced T cell-mediated immunity in two highly metastatic murine models of TNBC (Py8119 and 4T1) that are poorly responsive to PD-1 blockade [112], suggesting that αvβ6 integrin serves as a key regulator of the SOX4-driven immune escape pathway, although the precise mechanism remains unclear.

### 4.9. Glycosylation

Glycosylation is the process by which glycans are added to proteins, lipids, or other saccharides [113]. This is a major post-translational modification, which is regulated by glycosyltransferases and glycosidases. In cancer cells, the glycan biosynthetic pathway is deregulated, resulting in aberrant glycosylation [113]. Aberrant glycosylation is frequently associated with cancer progression, including tumorigenesis and metastasis [114]. Moreover, recent reports have indicated that altered glycosylation on ABC transporters and EGFR, as well as integrin, is associated with cancer chemoresistance [34,115].

Integrin heterodimers are transported to the cell surface after undergoing glycosylation at the Golgi apparatus. Integrin glycosylation is important for integrin functions, particularly with regard to cell adhesion, migration, and proliferation [116,117,118,119]. Our recent study has demonstrated that α6β4 integrin signaling confers doxorubicin resistance in melanoma MDA-MB435S cells and pancreatic cancer Panc-1 cells by suppressing caspase-3-mediated apoptosis [115]. Furthermore, the bisecting GlcNAc, which is downregulated in several drug-resistant cell lines [120,121], on β4 integrin negatively regulates the α6β4 integrin-mediated resistance to doxorubicin, suggesting that *N*-glycans on β4 integrin play a pivotal role in doxorubicin resistance and may serve as a potential biomarker of cancer chemoresistance (Figure 4b).

## 5. Potential Clinical Applications

Despite encouraging in vitro and in vivo preclinical results suggesting the potential efficacy of integrin-targeted cancer therapies, the clinical trial results have not met expectations [4,5]. The clinical trials have mainly involved antibodies (abituzumab, intetumumab, and etaracizumab) or peptides (cilengitide) targeting αv integrin. In addition, the anti-α5β1 integrin monoclonal antibody (volociximab), the small peptide antagonist of the α5β1 integrin ATN-161, and the non-peptide antagonist of RGD-binding integrin GLPG0187 have also been tested in clinical trials (Table 3). Unfortunately, these clinical trials targeting integrins did not show any treatment efficacy, likely due to the low drug delivery efficiency and unanticipated effects [5]. However, recent preclinical studies have indicated that novel approaches and improved treatment efficacy of anticancer drugs targeting integrins have the potential to overcome cancer drug resistance. In this section, we review recent preclinical findings and consider new opportunities for integrin-targeted drug development to overcome drug-resistant cancers. The section is divided into three parts: direct and indirect targeting and drug delivery.

### 5.1. Direct Integrin Targeting

The RGD mimetic cyclic peptide, cilengitide (also known as EMD 121974), directly inhibits αvβ3 and αvβ5 integrins, which are expressed on tumor cells and tumor endothelial cells. This fact led us to expect that cilengitide would inhibit tumor progression and tumor angiogenesis. Although cilengitide exhibited promising results in in vitro cell culture studies and in vivo preclinical animal studies, the phase 1 and 2 clinical trials with cilengitide in cancer therapy were unsuccessful [122]. An investigation into the cause of failure revealed that treatment with low (nanomolar) concentrations of cilengitide resulted in unanticipated pro-angiogenic effects through increased VEGFR2 protein expression via the post-transcriptional regulation and stimulation of VEGFR2 and αvβ3 integrin recycling, which promote endothelial cell migration [123]. Despite the systemic administration of high doses, tumors were exposed to the low-dose, pro-angiogenic effects of cilengitide because of the short plasma half-life of cilengitide, which ranges from 3 to 5 h, thereby explaining its limited efficacy in treating tumors [122]. These are considered to be the reasons for the failure of the clinical trials.

The discovery of the pro-angiogenic effects of low-dose cilengitide in tumors led to the exploration of an alternative use for low-dose cilengitide [122]. This alternative involves the use of low-dose cilengitide to enhance blood flow, which, in turn, leads to an increase in the drug delivery and intracellular uptake of chemotherapeutic drugs. In the KPC mouse model of pancreatic cancer and A549 human NSCLC models, the co-administration of low-dose cilengitide and the calcium channel blocker verapamil increased tumor angiogenesis, blood flow, and gemcitabine delivery, thereby reducing tumor progression and minimizing side effects and prolonging survival [124]. ProAgio, a designed protein recently developed by the Liu group, also uses a similar idea of improving drug delivery by eliminating leakage in tumor angiogenic vessels [125]. In TNBC tumors, the binding of ProAgio to αvβ3 integrin outside the ligand binding site induced apoptosis instead of blocking ligand binding in angiogenic endothelial cells, which highly expressed αvβ3 integrin, and reduced the leaky tumor vasculature [125]. The elimination of leaky tumor angiogenic vessels increased tumor blood perfusion, which consequently improved drug delivery and enhanced the doxorubicin-induced apoptosis in cancer cells [126]. Similarly, ProAgio improved intratumoral drug delivery and enhanced the efficacy of gemcitabine in PDAC. Alternatively, the depletion of cancer-associated pancreatic stellate cells, which are the primary source of collagen in PDAC tumors and exhibit high expression of αvβ3 integrin, via the targeting of αvβ3 integrin with ProAgio enhanced gemcitabine efficacy by reducing tumor collagen and altering gemcitabine metabolism in PDAC cells [127]. ProAgio is now in Phase I clinical trials for PDAC, TNBC, and other solid tumors (NCT05085548, NCT06182072, and NCT06460298).

Drugs directly targeting integrin can be used as carriers to deliver anti-cancer drugs. Treatment with clinical EGFR-TKIs (erlotinib, gefitinib, and lapatinib) resulted in the accumulation of αvβ3 integrin-positive cells, thereby leading to resistance to the aforementioned EGFR-TKIs in NSCLC cell lines (A549, H1975, and Lewis) [128]. The co-administration of EGFR-TKIs and cilengitide or a monoclonal antibody targeting αvβ3 (LM609) has been shown to efficiently reverse drug resistance in the αvβ3 integrin-positive EGFR-TKI-resistant cells [128,129]. In a mouse tumor model, as the tumors became resistant to erlotinib, αvβ3 integrin-negative HCC827 human EGFR-mutant lung tumors not only gained αvβ3 integrin but also became enriched for tumor-associated macrophages (TAMs) [129]. LM609 prevented acquired erlotinib resistance by exploiting TAMs to trigger antibody-dependent cellular cytotoxicity (ADCC). These findings suggest that an anti-αvβ3 integrin antibody could be a promising immunotherapeutic approach to redirect TAMs to function as tumor-killing cells for late-stage or drug-resistant cancers [129]. Taken together, these findings indicate that the cancer treatment strategy based on direct integrin targeting may still be a very powerful tool for cancer therapy, with approaches that differ from previous approaches that inhibit ligand binding to the αvβ3 integrin.

### 5.2. Indirect Integrin Targeting

In contrast to the direct targeting of integrins by binding therapeutic agents to integrins, the indirect targeting of integrins to overcome drug-resistant cancers can be achieved by inhibiting integrin signaling or suppressing integrin expression.

#### 5.2.1. Inhibition of Integrin Signaling

Certain integrin-positive patients with drug resistance may potentially benefit from a combination of an integrin signaling inhibitor and chemotherapy. The expression of αvβ3 integrin and the receptor tyrosine kinase AXL was found to be upregulated in erlotinib-resistant NSCLC cell lines (HCC827 ER and HCC4006 ER), as well as in tumor specimens obtained from NSCLC patients who had developed acquired resistance to erlotinib [130]. αvβ3 integrin promoted erlotinib resistance by upregulating AXL through the YAP pathway in erlotinib-resistant NSCLC cells, and the resistance to erlotinib was significantly inhibited by the AXL inhibitor R428 (bemcentinib). In addition, a novel compound, PAWI-2, inhibited αvβ3 integrin/KRAS/NF-κB signaling independently of KRAS [131]. PAWI-2 overcame erlotinib resistance in αvβ3 integrin-overexpressing pancreatic cancer FGβ3 cells, which exhibit CSC-like properties and anticancer drug resistance, by inducing G2/M cell cycle arrest during mitosis [131].

Tumor necrosis factor-α-induced protein 2 (TNFAIP2) is a downstream target of the transcription factor KLF5 and can interact with Rac1. α6β4 integrin activates Rac1 through TNFAIP2 and IQGAP1, which in turn confers resistance to the DNA damage-related drugs epirubicin, talazoparib, cisplatin, and olaparib in the TNBC cell lines HCC1806 and HCC1937 [132]. In addition, the α3β1/α11β1 integrins/Src/YAP pathway drove dual drug resistance to MAPK and PI3K/mTOR inhibitors in the melanoma cell lines SKMEL28 and WM2664 [46]. Thus, the α6β4 integrin/TNFAIP2/IQGAP1/Rac1 and α3β1/α11β1 integrins/Src/YAP pathways, as well as the αvβ3 integrin/YAP/AXL and αvβ3 integrin/KRAS/NF-κB pathways, may represent potential therapeutic targets to overcome drug resistance in cancer.

Cancers often develop resistance to chemotherapy-induced cell apoptosis during treatment, suggesting that targeting non-apoptotic pathways, such as pyroptosis, may be an alternative cancer treatment strategy [5,133]. In clinical practice, elevated αvβ5 integrin expression was associated with worse regression overall and progression-free survival in patients with TNBC treated with chemotherapy and patients with ovarian cancer or PDAC treated with gemcitabine [5,133]. αvβ5 integrin contributes to chemoresistance in cancer cells by suppressing chemotherapy-induced pyroptosis in pancreatic (TB32048 and Panc-1) and lung cancer (LLC and A549) cells [133]. Thus, targeting the αvβ5 integrin-mediated pyroptosis pathway may improve cancer drug resistance. Taken together, these findings suggest that integrin signaling plays a pivotal role in the chemoresistance of cancer cells. Table 4 provides a comprehensive overview of integrins involved in pro-survival and anti-apoptotic signaling in various chemoresistant cancer cells.

#### 5.2.2. Suppression of Integrin Expression

The suppression of integrin expression may be another indirect integrin-targeting strategy to overcome chemotherapeutic resistance. The αvβ3 integrin antagonist NDAT downregulated αvβ3 integrin expression in several cancer cell lines (pancreatic, breast, NSCLC, colorectal, hepatocellular, glioblastoma, bladder, and gastric cancers) in vitro and in vivo [134]. In an orthotopic pancreatic cancer model, NDAT suppressed the development of cisplatin resistance and the combination therapy of NDAT with cisplatin greatly inhibited tumor growth, accompanied by a significant increase in tumor necrosis compared to cisplatin alone [135]. Similarly, preincubation with a peptide derived from *Lentinus squarrosulus* prior to cisplatin treatment increased apoptosis in lung cancer cells through the suppression of integrin expression levels (αvβ1, αvβ3, αvβ5, and α5β1) and their downstream signals (FAK/Src/Akt) [136].

For indirect integrin targeting, miRNA can also be used to downregulate integrin expression. miR-150 and miR-142 suppress αv and β3 integrin expression, respectively. These miRNAs effectively inhibited αvβ3 integrin-dependent FAK activation and restored sensitivity to the HSP90 inhibitor AUY922 in the KRAS-mutated NSCLC cell lines A549 and H1944 [137]. miR-485-5p targeting keratin 17 suppressed α6β4 integrin expression and subsequent FAK/Src/ERK/β-catenin signaling in the OSCC cell lines C9IV3 and HSC3 [101]. Ectopic expression of miR-485-5p attenuated OSCC stemness properties and increased sensitivity to cisplatin or carboplatin in OSCC. miR-760 indirectly destabilizes the β1 integrin by targeting a novel RNA binding protein, Moloney leukemia virus 10 (MOV10). MOV10 has been shown to be upregulated in cancer cells compared to normal cells and to interact with and stabilize integrin β1 [138]. The destabilization of integrin β1 by miR-760 targeting MOV10 increased the sensitivity of the pancreatic cancer cell lines BxPC-3 and SW1990 to gemcitabine [138].

Despite the discovery of KRAS driver mutations nearly four decades ago, Ras has long been considered “undruggable”, lacking the potential for targeted treatment [139]. Recently, sotorasib and adagrasib were developed as covalent inhibitors of KRAS G12C and approved by the US FDA for the treatment of patients with NSCLC harboring the KRAS G12C mutation. However, it became clear that the effect was not sustained and that the therapeutic efficacy was inferior in other cancers, such as colorectal cancer. A recent study has shown that acquired resistance to sotorasib can be reversed by combining sotorasib with the proteasome inhibitor carfilzomib through the abrogation of α6β4 integrin and β-catenin expression and their downstream signaling [140]. These findings suggest that suppression of α6β4 integrin expression may be useful to overcome resistance to sotorasib.

### 5.3. Integrin-Mediated Drug Delivery

Compared to traditional chemotherapy, the targeted delivery of therapeutic drugs to cancer cells has been shown to be both efficient and accurate. Also, the targeted delivery of compounds to the tumor vasculature and tumor cells has the potential to improve anticancer therapy. Recent findings have demonstrated that integrins may represent a target for the delivery of compounds [141]. A bispecific antibody targeting the complex formed by the K^+^ channel hERG1 and the β1 integrin (scDb-hERG1-β1) exhibited high affinity binding to PDAC cell lines (Panc-1, MIA Paca-2, and BxPC3) but not to the human pancreatic ductal epithelial cells and pancreatic stellate cells [142]. In addition, the scDb-hERG1-β1 specifically penetrates the tumors of the Panc-1 mouse xenograft model. The combination of scDb-hERG1-β1 with a suboptimal dose of gemcitabine in mice implanted with Panc-1 cells demonstrated favorable therapeutic efficacy, minimal toxicity, and consequently, prolonged survival time of the mice [142]. Patients with TNBC who exhibit high β3 integrin expression tend to experience poorer clinical outcomes [51]. Targeted delivery of the αvβ3 integrin-specific quinolone nonpeptide micelle nanoparticle with rapamycin cargo to cells expressing activated αvβ3 integrin resulted in enhanced efficacy of docetaxel in the treatment of bone metastases of the PyMT-BO1 murine breast tumor cell line [51,143].

A novel tumor-penetrating peptide iRGD (CRGDK/RGPD/EC) can effectively and deeply deliver anticancer drugs into the tumor parenchyma, thereby enhancing the activity of anticancer drugs [144,145]. After intravenous injection, iRGD-abraxane (paclitaxel) accumulated in the tumor 11-fold more than non-targeted abraxane and approximately 4-fold more than the CRGDC peptide-abraxane and inhibited tumor growth in a BT474 mouse breast cancer xenograft model [146]. In KPC PDAC mouse models that express high levels of αvβ5 integrin, mice treated with the combination of iRGD and gemcitabine survived significantly longer than those treated with gemcitabine alone [147]. The iRGD peptide has a three-step tumor targeting mechanism: (1) the iRGD peptide binds to αvβ3 and αvβ5 integrins, but not to α5β1 integrin, on the tumor endothelium through the RGD motif; (2) the peptide is proteolytically cleaved to produce CRGDK/R and expose a C-end rule (CendR) motif (R/KXXR/K) within the TME; (3) the CendR motif binds to neuropilin-1 and -2 (NRP-1/2) upon activation to trigger tumor tissue penetration [146]. The iRGD peptide dispersed throughout the tumor tissue but two conventional RGD peptides (CRGDC and RGD-4C) accumulated only in and around tumor blood vessels [146]. A number of researchers are currently testing the effect of various modified versions of iRGD peptide in preclinical studies, suggesting that the iRGD peptide may serve as a therapeutic target to enhance the efficacy of anticancer drugs [144]. Given these findings, the use of iRGD peptides in the development of anticancer drugs may become a reality in the near future.

## 6. Conclusions

The emergence of drug resistance is a major challenge to the efficacy of cancer treatment. In this review, we have provided substantial evidence indicating that integrins play a central role in the development of drug resistance. The emergence of drug resistance in cancer is the result of a complex process involving integrin expression, ECM, drug transporters, EMT, cancer stemness, and glycosylation. Therefore, a deeper understanding of these mechanisms is required for the development of anticancer drugs and drug delivery systems to overcome drug resistance in cancer cells. Previous clinical trials targeting integrins have failed. However, recent preclinical studies have shown that novel approaches are possible by improving intratumoral drug delivery, using TAMs to induce ADCC, and suppressing integrin signaling and expression. In addition, enhancing treatment efficacy with tumor-penetrating drugs such as scDb-hERG1-β1 and iRGD peptides has great potential to overcome integrin-mediated drug resistance. Previous clinical trials have mainly targeted RGD-binding integrins. However, as discussed above, non-RGD integrins such as collagen-binding integrins and laminin-binding integrins also play an important role in the development of cancer drug resistance. As shown in Figure 2, ECM proteins can be recognized by multiple integrins. In other words, integrins exhibit redundancy, whereby the function of one integrin is compensated for by another. Given these considerations, combination therapy with multiple anti-integrin drugs may prove to be an effective approach. Therefore, the development of an anti-cancer therapy targeting collagen-binding integrins and laminin-binding integrins would be useful. It is hoped that the development of integrin-targeted therapeutics against drug-resistant cancers will facilitate the overcoming of drug resistance in cancer in the future.

## Figures and Tables

**Table 1:** Cell adhesion-mediated drug resistance (CAM-DR) in solid tumors.

Integrin Subunit	Tumor Type	Biological Functions	Signaling Pathways	References
β1	NSCLC (A549)	Resistance to cisplatin		[14]
	OSCC (Ca9-22, SAS) Breast (MCF-7, MDA-MB231) Ovarian (W1)	Inhibits 5-FU-induced apoptosis Resistance to cisplatin, mitoxantrone Resistance to cisplatin	ILK/Akt/NF-κB FAK/PI3K/Akt/MAPK mTOR	[15] [16,17] [18]
α6β1	Bone-metastatic CRPC (LuCaP, C4-2)	Promote resistance to PI3K inhibitors by reducing oxidative stress and preventing cell death		[19]
α2β1	Breast (MDA-MB231) Melanoma (MDA-MB435S)	Inhibits paclitaxel-induced apoptosis Inhibits paclitaxel-induced apoptosis	PI3K/Akt PI3K/Akt	[20] [20]
α5β1	Breast (MDA-MB231)	Inhibits paclitaxel-induced apoptosis	PI3K/Akt	[20]
	Melanoma (MDA-MB435S)	Inhibits paclitaxel-induced apoptosis	PI3K/Akt	[20]

NSCLC, Non-small cell lung cancer; OSCC, Oral squamous cell carcinoma; CRPC, Castration-resistant prostate cancer.

**Table 2:** Overexpressed integrin subunits and their expression levels in tumor tissues compared to normal tissues.

Integrin Subunit	Tumor Tissues (Expression Level)
α1	GBM (2.86), HNSCC (1.54), KIRC (0.77)
α2	BLCA (0.87), CESC (2.84), CHOL (6.25), HCC (2.32), LUAD (1.79), LUSCC (1.21), STAD (1.82)
α3	BLCA (1.11), CHOL (5.3), HNSCC (1.83), KIRC (0.48), KIRP (1.38), LUAD (0.7), STAD (0.86)
α4	BRCA (0.57), CHOL (1.14), GBM (3.2), HNSCC (1.04), KIRC (1.62), PCPG (2.35), STAD (1.01)
α5	BRCA (0.27), CHOL (2.45), GBM (2.95), HNSCC (2.53), KIRC (1.88), HCC (1.15)
α6	CHOL (2.51), HNSCC (2.01), KICH (1.53), KIRC (0.33), HCC (2.02), LUSCC (1.36), PAAD (1.45), PCPG (2.76), STAD (1.44)
α7	GBM (1.58), KICH (0.68), KIRC (0.83), HCC (1.1), LUAD (0.34)
α8	CHOL (1.56)
α10	CHOL (1.89), KIRC (0.95), PRAD (0.38)
α11	BRCA (1.08), CHOL (2.59), HCC (1.75), LUAD (3.1), LUSCC (1.88), READ (1.69), STAD (2.22)
αD	BRCA (0.48), CHOL (1.55), KICH (2.83), KIRC (4.58), PRAD (1.23), STAD (1.65)
αE	BLCA (0.54), CHOL (2.24), HNSCC (0.39), STAD (0.78)
αL	BRCA (1.04), GBM (1.43), HNSCC (0.75), KIRC (2.63), KIRP (1.05), HCC (0.37), STAD (0.8)
αM	BRCA (0.48), CHOL (3.45), GBM (1.43), KIRC (1.8), KIRP (1.58), HCC (0.87), STAD (1.22)
αV	CHOL (3.36), GBM (0.93), HNSCC (1.23), HCC (1.42), LUAD (1.2), LUSCC (0.81), STAD (0.68)
αX	BRCA (0.89), CESC (1.84), CHOL (1.75), HNSCC (1.46), KICH (1.41), KIRC (3.33), KIRP (3.02), HCC (0.62), PRAD (0.68), STAD (2.36)
αIIb	BLCA (1.69), BRCA (0.72), CHOL (3.13), HNSCC (1.83)
β1	CHOL (2.71), GBM (1.66), HNSCC (0.74), KIRC (0.27), HCC (0.59), STAD (0.63)
β2	BRCA (0.75), CHOL (1.53), GBM (1.78), HNSCC (0.97), KIRC (2.37), KIRP (1.55), STAD (1.35)
β3	GBM (2.83), PCPG (3.62)
β4	BLCA (1.01), CESC (2.33), CHOL (5.88), GBM (1.61), HNSCC (1.4), KIRC (0.52), HCC (2.07), LUAD (2.03), LUSCC (2.57), PAAD (1.74)
β5	BLCA (0.6), BRCA (0.27), CHOL (2.32), HNSCC (0.78), HCC (0.89), LUAD (0.69), LUSCC (0.52), STAD (0.67)
β6	BLCA (1.12), CESC (5.91), CHOL (6.14), GBM (1.21), HNSCC (1.39), STAD (1.59)
β7	BRCA (0.76), CESC (2.57), CHOL (1.27), KICH (0.89), KIRC (0.58), HCC (0.46), LUAD (0.75)
β8	CHOL (4.55), GBM (1.24), KIRP (1.63), LUAD (1.67), LUSCC (2.14), READ (0.91), STAD (1.33)

Integrin expression data were taken from reference [32]. The integrin subunit data used in this reference were extracted from the total RNA-seq expression dataset in the TCGA database, normalized, and calculated. All datasets without normal samples were discarded. Expression levels are log2 fold change values. Urothelial Bladder Carcinoma (BLCA), Breast-Invasive Carcinoma (BRCA), Cervical Squamous Cell Carcinoma and Endocervical Adenocarcinoma (CESC), Glioblastoma Multiforme (GBM), Head and Neck Squamous Cell Carcinoma (HNSCC), Kidney Chromophobe (KICH), Kidney Renal Cell Carcinoma (KIRC), Kidney Renal Papillary Cell Carcinoma (KIRP), Liver Hepatocellular Carcinoma (HCC), Lung Adenocarcinoma (LUAD), Lung Squamous Cell Carcinoma (LUSCC), Pancreatic Adenocarcinoma (PAAD), Paraganglioma and Pheochromocytoma (PCPG), Prostate Adenocarcinoma (PRAD), Rectum Adenocarcinoma (READ), Stomach Adenocarcinoma (STAD).

**Table 3:** Clinical studies of integrin-targeting therapies.

Generic Name	Modality	Integrin Target	Cancer Types	Number of Patients Enrolled	Clinical Trials Gov Identifiers (Status)
Abituzumab	Antibody	αv	CRC Prostate	216 180	NCT01008475 (Completed) NCT01360840 (Completed)
Intetumumab (CNTO 95)	Antibody	αv	Stage 4 melanoma Metastatic prostate	144 131	NCT00246012 (Completed) NCT00537381 (Completed)
Volociximab (M200)	Antibody	α5β1	NSCLC Metastatic pancreatic Ovarian Ovarian, Peritoneal Metastatic melanoma Metastatic RCC	33 40 16 138 40 48	NCT00654758 (Completed) NCT00401570 (Completed) NCT00516841 (Terminated) NCT00635193 (Completed) NCT00099970 (Completed) NCT00100685 (Terminated)
GLPG0187	Small molecule	α5β1/αv	Solid tumors	20	NCT01313598 (Completed)
Cilengitide (EMD121974)	Cyclic peptide	αvβ3, αvβ5	Glioblastoma HNSCC Prostate NSCLC	545 184 16 232	NCT00689221 (Completed) NCT00705016 (Completed) NCT00121238 (Completed) NCT00842712 (Completed)
ATN-161	Small molecule	α5β1	Advanced RCC Glioma	36 82	NCT00131651 (Terminated) NCT00352313 (Completed)
Etaracizumab (MEDI-522, Abegrin) (derived from LM609)	Antibody	αvβ3	Metastatic melanoma Kidney Prostate	110 5 150	NCT00066196 (Completed) NCT00684996 (Terminated) NCT00072930 (Completed)
CEND-1	iRGD peptide	αv	Metastatic pancreatic	30	NCT03517176 (Completed)
7HP349	Small molecule	αLβ2, α4β1	Normal healthy male	60	NCT04508179 (Completed)
SGN-B6A	Antibody-drug conjugate	β6	Advanced solid tumors	824 (estimated)	NCT04389632 (Recruiting)
OS2966	Antibody	β1	High-grade glioma	7	NCT04608812 (Terminated)
E-7820	Small molecule	α2β1	Solid tumors	45	NCT01773421 (Completed)
MK-0429	Small molecule	αvβ3	Metastatic prostate	29	NCT00302471 (Completed)
ProAgio	Cytotoxic protein	αvβ3	PDAC, other solid tumors PDAC TNBC	58 (estimated) 28 (estimated) 51 (estimated)	NCT05085548 (Recruiting) NCT06182072 (Recruiting) NCT06460298 (Recruiting)

PFS ns different represents progression-free survival that is not significantly different.

**Table 4:** Integrins involved in pro-survival and anti-apoptotic signaling in chemoresistant cancer cells.

Signal	Integrin
PI3K/Akt/Src/mTOR Ras/Raf/MAPK/ERK NF-κB JAK/STAT YAP Wnt/β-catenin	β1, α2β1, α3β1, α5β1, α7β1, α10β1, α11β1, α6β4, αvβ3, αvβ5, αvβ6, αvβ8 β1, α2β1, α6β4, αvβ3 β1, αvβ3, αvβ8 αvβ6 β1, α3β1, α11β1, αvβ3 β1, α6β4, αvβ3
JNK	α3β1
